# Treatment outcomes in a rural HIV clinic in South Africa: Implications for health care

**DOI:** 10.4102/sajhivmed.v17i1.414

**Published:** 2016-05-27

**Authors:** Olufemi B. Omole, Mary-Anne M.L. Semenya

**Affiliations:** 1 Department of Family Medicine, University of the Witwatersrand, South Africa

## Abstract

**Objective:**

To assess the treatment outcomes of an HIV clinic in rural Limpopo province, South Africa.

**Methods:**

A retrospective cohort study involving medical records review of HIV-positive patients initiated on antiretroviral treatment (ART) was conducted from December 2007 to November 2008 at Letaba Hospital. Data on socio-demographic characteristics, CD4 counts, viral loads (VLs), opportunistic infections, adverse effects of treatment, hospital admissions, and patient retention at 6, 12, 24, and 36 months on ART were collected. Analysis included descriptive statistics, chi-square and *t*-tests.

**Results:**

Of 124 patient records sampled, the majority of patients were female (69%), single (49%), unemployed (56%), living at least 10 km from the hospital (52.4%), and were on treatment at 36 months (69%). Approximately 84% of patients achieved viral suppression (VLs < 400 copies/mL) by 6 months of ART and the mean CD4 count increased from 128 at baseline to 470 cells/mm^3^ at 24 months. There was a mean weight gain of 5.9 kg over the 36 months and the proportion of patients with opportunistic infections decreased from 54.8% (*n* = 68) at baseline to 15.3% (*n* = 19) at 36 months. Although the largest improvements in CD4, VLs, and weights were recorded in the first 6 months of ART, viral rebound became evident thereafter. Of all variables, only age < 50 years and being pregnant were significantly associated with higher VLs (*p* = 0.03).

**Conclusion:**

Good treatment outcomes are achievable in a rural South African ART clinic. However, early viral rebound and higher VLs in pregnancy highlight the need for enhanced treatment adherence support, especially for pregnant women to reduce the risk of mother to child transmission.

## Introduction

South Africa currently has the highest HIV infection burden in the world, with an estimated 6.4 million people infected by the virus in 2012.^1^ Responding to high HIV-related morbidity and mortality, the South African government implemented a nationwide roll-out of an antiretroviral treatment (ART) programme in public health facilities in 2004. This has resulted in the largest treatment programme globally, with more than 2.4 million people on treatment by mid-2013.^2^

South Africa has a significant rural population with some of the highest HIV burdens reported in this population.^3^ In addition, the South African rural context is a unique and vulnerable population, often characterised by poor socio-economic conditions, limited access to healthcare services, limited available clinical support, and poor healthcare resources.^4^ All of these issues have been reported to adversely affect healthcare provision, resulting in poorer health outcomes in the rural areas compared to urban South Africa.^4^

A decade and more after the nationwide roll-out of the ART programme in public health facilities in South Africa, several studies have reported good treatment outcomes, including significant reductions in mortality and morbidity among HIV-positive patients on ART.^5,6,7,8^ Most of these studies were conducted in peri-urban and tertiary centres, with only a few originating from the rural or resource-constrained areas.^9,10,11^ Although these few rural studies have also reported favourable outcomes, all except the report of Barth et al. (2011) have been conducted in areas other than the Limpopo province. In order to build a more robust and more representative body of knowledge on treatment outcomes of ART programmes in rural South Africa, more recent data are needed from other rural and resource-constrained areas of South Africa. The aim of this study was therefore to assess the treatment outcomes of a public service HIV clinic in the predominantly rural province of Limpopo. This article presents the findings of this study and discusses the implications for HIV care.

## Methods

### Design

Retrospective cohort study involving review of patients’ medical records.

### Study setting and study participants

This study was conducted at Nyeleti Clinic, Letaba Regional Hospital (LRH) between November 2011 and August 2012. LRH is situated in Mopani district, Limpopo province and during the study period, served as a referral centre for six district hospitals, one specialised hospital, two immediate community health centres, and 18 primary care clinics. Mopani district is home to approximately 1.1 million people, 81% who live in rural dwellings and 77% who live below the poverty line.^12^ Though situated in a specialist hospital, HIV care at Nyeleti Clinic is nurse-driven and supported by one or two medical officers allocated on a daily basis from LRH.

During the period under review, standard treatment regimen^13^ for HIV in the South African public healthcare included stavudine (d4T), lamuvidine (3TC), and efavirenz in regimen 1a; stavudine (d4T), lamuvidine (3TC), and nevirapine (NVP) in regimen 1b; and zidovudine (AZT), didanosine (ddl), and lopinavir/ritonavir (LPV/r) in regimen 2.

The required sample size was calculated to be 124 files. In order to sample patient records, all 687 adult patients who were initiated on ART at the clinic between December 2007 and November 2008 were entered into a register in the order of the dates of enrolment into treatment. The first patient was randomly selected by the second author from the register. Thereafter, although a sampling fraction of 5 (687/124) would have yielded the required sample size, the researchers sampled every third patient to accommodate potential missing files. If a sampled patient’s file did not meet the inclusion criteria, the next patient on the register was selected. The systematic sampling continued until the required sample size was achieved. To be included a patient had to be 18 years or older at the time of ART initiation and had to have been initiated on ART at LRH. Patients whose files could not be found in the archive were excluded.

### Measurements

Data were extracted from patients’ files onto a data collection sheet by the second author. Identifying data were not collected but a coding system was used to enable the researchers to trace the patient’s files if there was a need and also to avoid reselection of files. Data extracted included: patient’s socio-demographic characteristics (age, sex, place of residence, level of education, employment, and marital status); treatment outcomes (CD4 counts, VLs, presence of opportunistic infections, hospital admissions, weight, and adverse effects recorded at 6, 12, 24, and 36 months); and whether the patient was still attending the ART clinic at 6, 12, 24, and 36 months (Table 1).

**TABLE 1:** Patients’ socio-demographic characteristics and treatment regimen.

Variable	%†	*N*	Mean ± s.d.
**Age**	-	-	41 years
**Sex**	-	-	-
Male	69	85	-
Female	28	35	-
**Marital status**	-	-	-
Single	49	61	-
Married	33	41	-
Divorced	6	7	-
Widowed	5	6	-
Unspecified	7	9	-
**Employment status**	-	-	-
Unemployed	56	69	-
Employed	12	15	-
Unspecified	32	40	-
**Educational attainments**	-	-	-
Unspecified	47	58	-
None/primary	22	27	-
Secondary	28	35	-
Tertiary	3	4	-
**Distance between residence and hospital**	-	-	-
< 10 km	47.6	59	-
10 km – 20 km	32	40	-
> 20 km	20	25	-
**Clinical regimen and status at baseline**	-	-	-
Regimen 1a	97	120	-
Regimen 1b	3	4	-

† Due to missing data, ‘*n*’ is not always equal to 124.

### Pilot study

A pilot study was conducted at the ART clinic of a nearby district hospital with 25 patient files. This assisted in checking whether the study was feasible and the ease of use of the data collection tool.

### Analysis and definition of outcomes

The data collected were entered into Epi Info^TM^ version 6 for descriptive analysis and thereafter imported into STATA version 9.0 for testing of associations between socio-demographic characteristics and treatment outcomes. Statistical significance was deemed to exist if *p* value was less than 0.05. Main outcome measures analysed for at baseline, 6, 12, 24, and 36 months after initiation on ART included:

- The proportion of patients with viral suppression – defined as median VL < 400 copies/mL.
- The proportion of patients with immune recovery – defined as median CD4 count > 200 cells/mm^3^.
- The mean weight gain, expressed in kg.
- The proportion of patients admitted to hospital.
- The proportion of patients with opportunistic infections.
- The proportion of patients who were still attending the HIV clinic at LRH. Patients who did not attend the HIV clinic for more than 3 months after the last visit without known reasons were considered lost to follow up. Those who missed their clinic visit for up to 3 months were classified as defaulters.

## Ethical consideration

The research protocol was approved by the Human Research and Ethics Committee at the University of Witwatersrand (Clearance certificate number M110485). Permission to conduct the study was granted by both the Limpopo province provincial research committee and the chief executive officer of LRH.

## Results

In total, 124 patients’ files were sampled. The mean sample age was 41 years with the majority being female (69%), single (49%), unemployed (56%), living 10 km or farther from the hospital (52%), and initiated on regimen 1a of the South African ART programme at baseline (97%). Forty-two patients (34%) had concurrent clinical conditions and 20 (24% of women) became pregnant during the period under review (Table 2).

**TABLE 2:** Concurrent clinical conditions.

Types of clinical conditions (*n* = 42)†	%	*n*
Pregnancy (4 patients fell pregnant twice during the period under consideration)	16.1	20
Arthritis	10.5	13
Hypertension	4.0	5
COPD	1.6	2
Diabetes	0.8	1
Others: cancer, peptic ulcer disease, depression, cardiac, obesity, epilepsy	4.8	6

† Some participants had more than one concurrent clinical condition.

At baseline, the median CD4 count was 134 cells/mm^3^ with 93% of patients having CD4 counts of 200 cells/mm^3^ or less. Median CD4 counts increased progressively with time after ART initiation to 408 cells/mm^3^, the largest increases occurring in the first 6 months. There were statistically significant differences between the median CD4 counts at baseline compared to all other time periods (*p* < 0.05) (Table 3).

**TABLE 3:** CD4 counts and viral loads.

Variables	Ranges	Baseline		6 months		12 months		24 months	
			
		*N*	%	*n*	%	*N*	%	*n*	%
CD4 count†	< 50	19	16	2	2	3	3	2	3
	50–100	27	23	11	11	4	4	4	5
	101–200	64	54	16	15	16	16	7	9
	> 200	9	7	73	71	78	77	65	83
	Mean CD4	128	-	310	-	380	-	470	-
	Median CD4	134	-	289	-	361	-	408	-
**Total (*n*)**		**119**	**-**	**102**	**-**	**101**	**-**	**78**	**-**
Viral load (VL)‡	< 50	5	7	68	67	61	61	50	63
	50–400	9	12	17	17	18	18	10	13
	401–5000	20	26	16	16	12	12	7	9
	5000+	43	55	-	-	9	9	12	15
	Median VL	99 358	-	3597	-	20 599	-	5895	-
**Total (*n*)**		**77**	**-**	**101**	**-**	**100**	**-**	**79**	-

† CD4 Count: Baseline v/s 6 months (*p* < 0.05); baseline v/s 12 months (*p* < 0.005); baseline v/s 24 months (*p* < 0.005);

‡ Viral load: Baseline v/s 6 months (*p* < 0.05); baseline v/s 12 months (*p* < 0.005); baseline v/s 24 months (*p* < 0.005).

At baseline, the median VL was 99 358 copies/mL with the majority of patients (81%) having VLs greater than 400 copies/mL. However, by 6 months of ART, 84% had achieved viral suppression. After 6 months of ART, viral rebound surfaced, evidenced by a significant increase in the proportion of patients with VLs > 5000 copies/mL (*p* < 0.005).

The prevalence of opportunistic infections decreased from 58.4% at baseline to 15.3% at 36 months after initiation of ART. The most prevalent opportunistic infections were oral thrush (31%) and pulmonary tuberculosis (28%), with tuberculous meningitis emerging after 12 months on ART. Only 7.2% (nine) patients were admitted to hospital during the entire study period, with the number of admitted patients decreasing with time on ART.

Patients’ weights increased with time on ART, with the most significant increases occurring between baseline and 6 months after ART initiation (Mean weight 57.1 kg at baseline vs. 62.1 kg at 6 months of ART; *p* = 0.01).

Although the number of patients retained in treatment progressively decreased, 69% (*n* = 85) were still attending the ART clinic at 36 months. Of the 39 patients that were no longer in the treatment programme, 33 were accounted for – 22 (67%) were transferred to other hospitals or feeder PHC clinics, 4 (12%) defaulted treatment, 3 (9%) were lost to follow up, and 4 (12%) had died.

On association testing, patients who became pregnant at any time after baseline were significantly more likely to have higher VLs than those who did not. Compared to others, patients who were older than 50 years of age were significantly more likely to have viral suppression after 6 months of ART (*p* = 0.03). There was no significant association between socio-demographic characteristics and retention in care (Tables 4 and 5).

**TABLE 4:** Patient retention in care at time periods.

Time	Patient retention (%)	*n*
Baseline	100	124
6 months	99.2	123
12 months	93.6	116
24 months	89.5	111
36 months	86.3	107

**TABLE 5:** Associations of viral load and pregnancy status.

Timelines	Ranges	Pregnant				*p*

		No		Yes		
	
		*N*	%	*N*	%	
Baseline	< 50	4	10	0	0	0.46
	50–400	7	17	0	0	
	401–5000	12	29	4	40	
	> 5000	18	44	6	60	
6 months	< 50	35	69	9	53	0.02
	50–400	11	22	1	6	
	401–5000	5	10	7	41	
	> 5000	0	0	0	0	
12 months	< 50	38	73	5	29	0.002
	50–400	9	17	4	24	
	401–5000	4	8	5	29	
	> 5000	1	2	3	18	
24 months	< 50	29	69	5	42	0.03
	50–400	3	7	4	33	
	401–5000	2	5	2	17	
	> 5000	8	19	1	8	

## Discussion

This study is one of few that have investigated treatment outcomes in rural HIV clinics in South Africa and found that good treatment outcomes such as viral suppression, improved CD4 count, weight gain, and high patient retention in care are achievable in rural settings. These good outcomes are similar to those reported in peri-urban and well-resourced settings^5,7,14^ and suggest that the resource constraints of the South African rural setting may not be a barrier to rendering quality HIV care in this context.

The initiation of ART resulted in significant increases in the CD4 counts and viral suppression, in line with expected recovery in patients’ immunity.^15^ However, viral rebound that became evident within 12 months of ART is a cause for serious concern and possibly indicates poor treatment adherence or the development of resistance to treatment.^5,16^ This highlights the need for adherence support; close monitoring of laboratory and clinical parameters; and regular drug treatment reviews, especially among pregnant women, among whom this is crucial for the prevention of mother to child transmission.^17,18^ Electronic or paper-based prompts could be used to compel healthcare providers to timeously request due tests, review results, and make treatment changes when necessary. Healthcare providers’ responses to these prompts need to be monitored, and in the rural setting where doctors are in short supply and most programmes nurse-driven, this responsibility could be placed on the supporting doctor, as part of the clinical oversight and quality assurance functions. Apart from healthcare providers’ roles, patients also need to be empowered to play active roles in the management of their own condition by gaining better understanding of their disease, the benefits of treatment adherence, the necessity for close monitoring, the need to partner with healthcare providers in managing their condition, and the skills needed for coping with life and their conditions.

The vast majority of patients in this study presented with CD4 count less than 200 cells/mm^3^ and VLs higher than 400 copies/mL at baseline, suggesting late presentations. This behaviour has been associated with increased mortality, especially at CD4 < 50 cells/mm^3^,^19^ and highlights the need to promote early enrolment in the ART programme. Interventions such as the coupling of point of care (spot) CD4 count testing with all positive results for HIV tests could be used to facilitate early enrolment and has the potential to eliminate the unnecessary wait for results of laboratory-based CD4 counts that is often a barrier to immediate initiation of ART.^20^ Spot CD4 count testing can also significantly reduce the number of patients lost to follow up as a result of deferring CD4 count testing to later dates (when morbidity associated with advanced disease state usually forces the patient to seek healthcare).

Although more than half of patients in this study lived > 10 km from the ART clinic, 69% were still retained in care at 36 months, contradicting reports that distance from healthcare facilities predicts poor clinic attendance and healthcare outcomes in rural South Africa.^4,17^ The finding of high patient retention concurs with figures reported in other studies^5,6,7,21^ and may suggest that socio-economic challenges of the rural areas may not be barriers to achieving good treatment outcomes in this setting. Patients may indeed perceive that it is more beneficial to continue to attend HIV treatment in spite of the costs and pains of doing so.

In this study, improvements in laboratory parameters were corroborated by improvement in clinical and anthropometric measures. There were reductions in the prevalence of opportunistic infections and hospital admissions, and significant weight gain with time on ART. These improvements confirm reports from other settings^5,7,12^ and have clinical and financial implications, especially in terms of reduced costs and mortality associated with protracted hospital admissions.^22^ The emergence of TB meningitis several months after ART initiation is however a serious cause for concern and calls for a high index of clinical suspicion of TB among patients on ART. In addition to this, VLs and CD4 counts need to be monitored regularly, especially because high VLs and low CD4 are two strong predictors of TB disease progression among patients on ART.^23^

Weight gain (especially in the first 6 months) is most likely a result of many interwoven issues that may include reversal of HIV-associated wasting, reduction in prevalence of opportunistic infections, better nutrient absorption following decrease in the episodes of recurrent gastroenteritis, and a general improvement in the quality of life that enables HIV-infected people to better care for their own nutritional needs. Viewed through a psychosocial lens, weight gain could boost patient’s morale and confidence in the ART, reduce stigma associated with wasting, and may be used as motivation to re-enforce treatment adherence. However, weight gain needs to be monitored closely considering that obesity and lipodystrophy have been associated with metabolic syndrome, diabetes, and hyperlipidaemia.^24^

Although it was not possible to determine whether pregnancies were planned or not, the finding that 23.5% of women in this study became pregnant may confirm reports that risky and unsafe sexual behaviours tend to increase among HIV-infected people in the immediate period after ART initiation.^25^ Although these behaviours are not well understood, it is possible that improved health status that follows ART initiation may give a false ‘sense of security’ that promotes risk taking among HIV-infected patients, especially within steady relationships. A priority task in the healthcare provider-patient relationship is therefore to promote and sustain the adoption of safe sexual behaviours through on-going counselling and the provision of sexual health services (including family planning) as integral parts of HIV care to adult women. This is more so that pregnancy may have a non-lasting but deleterious effect on the clinical course and immunological outcomes among women on ART.^26^ Given the association between being pregnant and high VLs in this study, it is important to pay close attention to treatment adherence and laboratory indices monitoring in order to prevent mother to child transmission of the HIV infection.

Women constituted the majority of patients in this rural, facility-based study, confirming previous reports that women are disproportionately affected by HIV in South Africa.^4^ Interventions designed for HIV care in South Africa should therefore take cognisance of this and target women for testing and increased uptake into ART programmes. However, because men in this study also presented with lower mean CD4 counts and higher VLs at enrolment – two measures that predict poor treatment outcomes,^17^ they also need to be targeted for early uptake into HIV testing and ART.

This study is subject to some limitations. The use of record review makes the study susceptible to information bias from incomplete and missing data. The under-ascertainment of deaths could also have resulted in misclassification of a significant proportion of dead patients as being lost to follow up. The use of a single research site and a small sample size is a significant limitation on the ability to generalise the study findings nationally and to other rural areas in South Africa. Notwithstanding these limitations, this study is one of few that report treatment outcomes in rural, less-resourced, and primary care-based HIV clinics in South Africa and provides useful insights into the treatment outcomes of HIV care in this setting.

## Conclusion

Good treatment outcomes and high patient retention in care are achievable in a rural South African ART clinic. Viral rebound that emerged soon after initial viral suppression and the higher VLs in pregnancy, call for interventions that strengthen treatment adherence, particularly for PMTCT. Late presentations with low CD4 count and high VLs signify the importance of promoting early enrolment into HIV care and the need to implement spot CD4 count testing at diagnosis. Given the study limitations, larger and better designed studies are needed to validate these findings.
